# Craniotomy Complexity and Outcomes in Exoscope-Assisted Cranial Surgery: A Single-Center Retrospective Analysis

**DOI:** 10.3390/brainsci15101060

**Published:** 2025-09-29

**Authors:** Salvatore Cardali, Alfredo Conti, Domenicantonio Collufio, Domenico Matalone, Antonio Morabito, Francesco Messineo, Giuseppe Ricciardo, Giovanni Raffa, Giada Garufi

**Affiliations:** 1 Department of Neurosurgery, Azienda Ospedaliera Papardo, University of Messina, 98158 Messina, Italy; 2 Department of Biomedical, Dental and Morphological and Functional Imaging, University of Messina, Via Consolare Valeria, 98125 Messina, Italy; giovanni.raffa@unime.it; 3 Department of Neurosurgery, IRCCS Istituto delle Scienze Neurologiche di Bologna, 40139 Bologna, Italy; 4 Dipartimento di Scienze Biomediche e Neuromotorie (DIBINEM), Alma Mater Studiorum Università di Bologna, Via Altura 3, 40123 Bologna, Italy

**Keywords:** exoscope, craniotomy complexity, brain tumor, neuroncology, technology in neurosurgery

## Abstract

Objective: The exoscope is an emerging digital visualization technology in neurosurgery that provides high-definition 3D 4k magnified views of the surgical field on external monitors, promoting improved ergonomics and enhanced team involvement. This study presents a single center experience of 26 patients undergoing brain tumor resection using the Olympus Orbeye exoscope with surgical approaches of different complexities and provides a review of the current literature on exoscopic adoption in neurosurgical oncology. Methods: We retrospectively reviewed clinical, surgical, and outcome data from a consecutive series of 26 patients who underwent brain tumor resection with the ORBEYE exoscope. Metrics analyzed included extent of resection, surgical technique, and complications in two different complex scenarios: superficial and deep lesions. Results: In our institutional case series, use of the exoscope enabled gross total or subtotal resection in all the patients, with a surgical complication rate comparable to that reported for operative microscopes (14.3–23.1%), which was stated to be non-significant and independently correlated to the use of the exoscope. No device-related adverse events were observed, and postoperative neurological outcomes were in line with the overall survival pathological examination of the lesion treated. Conclusions: In this cohort, the exoscope enabled the safe and effective resection of superficial and deep lesions with outcomes comparable to those historically reported with operating microscopes. Gross total resection rates were high in the superficial cohort and substantially higher than in the deep cohort, while complication rates did not differ significantly between groups. Future prospective studies with long-term follow-up are needed to assess oncological outcomes and define the optimal role of exoscopic technology in neurosurgical oncology.

## 1. Introduction

The surgical management of brain tumors has undergone significant technological evolution over the past decades, with the introduction of advanced visualization systems representing a major milestone in neurosurgical practice. Traditional operating microscopes, while providing excellent magnification and illumination, present inherent limitations, including restricted positioning flexibility, ergonomic challenges for surgeons, and limited integration with modern digital workflows. The introduction of exoscope technology has addressed many of these limitations, offering high-definition 3D visualization on external monitors while maintaining the precision required for complex neurosurgical procedures [1,2]. Exoscopes provide several theoretical advantages over conventional microscopy, including improved ergonomics through natural head positioning, enhanced surgical team visualization, better integration with navigation systems, and the potential for superior depth perception through advanced 3D imaging [3]. However, the clinical impact of these technological advantages on surgical outcomes, particularly in relation to the complexity of craniotomy approaches and the extent of tumor resection, remains incompletely characterized in the literature [4].

The relationship between surgical complexity and clinical outcomes in brain mass surgery is multifaceted. Craniotomy complexity is influenced by numerous factors, including tumor location, size, proximity to eloquent brain areas, and the specific surgical approach required. Deep-seated lesions often necessitate more complex surgical corridors, potentially increasing operative time, technical difficulty, and the risk of complications. Conversely, superficial lesions may allow for more straightforward surgical approaches but can present their own challenges depending on their relationship to critical cortical areas. Gross total resection (GTR) remains the gold standard for most brain tumor pathologies, in particular for glioma surgery, with extensive literature demonstrating its correlation with improved survival outcomes and reduced recurrence rates [5,6]. However, achieving GTR must be balanced against the risk of neurological morbidity, particularly when tumors are located in eloquent brain regions or require complex surgical approaches. The extent of resection is influenced by multiple factors, including tumor characteristics, surgical technique, visualization quality, and the surgeon’s ability to distinguish tumor from normal brain tissue [7].

The integration of advanced visualization technologies, such as exoscopes, into neurosurgical practice has the potential to influence both the feasibility of achieving GTR and the associated complication rates [8]. Enhanced visualization may facilitate more precise tumor–brain interface identification, potentially improving resection rates while minimizing damage to surrounding normal tissue [7]. However, the learning curve associated with new technology and potential changes in surgical workflow must also be considered when evaluating clinical outcomes [4,9]. Previous studies examining exoscope technology in neurosurgery have primarily focused on technical feasibility, surgeon satisfaction, and basic safety parameters [10,11] or analyzed the feasibility of the exoscope assisted surgery on nonmicrosurgical procedures, such as the evacuation of subdural hematomas or peripheral nerve neurolysis. Limited data exists regarding the relationship between exoscope use and surgical outcomes across different levels of craniotomy complexity. Understanding this relationship is crucial for optimizing patient selection, surgical planning, and outcome prediction in the era of advanced surgical visualization. The present study aims to evaluate the relationship between craniotomy complexity and surgical outcomes in brain tumor patients operated on using exoscope technology. Specifically, we sought to determine whether craniotomy complexity correlates with the extent of resection and complication rates and whether outcomes differ between superficial and deep lesions when utilizing exoscope visualization. By systematically analyzing these relationships, we aim to provide evidence-based insights into the clinical utility of exoscope technology across the spectrum of neurosurgical complexity. Our hypothesis was that while deep lesions would require more complex craniotomy approaches, the advanced visualization capabilities of the exoscope would maintain consistent surgical outcomes across different complexity levels, potentially demonstrating the technology’s ability to mitigate some of the traditional challenges associated with complex neurosurgical approaches.

## 2. Materials and Methods

This retrospective study was conducted with institutional approval, and the Institutional Review Board (IRB) waived the requirement for formal ethics review due to the retrospective nature of the study. All the patients gave informed consent for the procedure and for the treatment of the data. The statistical analysis used appropriate non-parametric statistical methods given the small sample size, ordinal complexity scores, and binary outcomes, with a significance value stated at *p* < 0.01 (Phyton 3.13.5, Python Software Foundation, Beaverton, OR, USA). This retrospective study analyzed 26 consecutive patients who underwent brain mass resection using exoscope technology (Orbeye Surgical Visualization System, Olympus Corporation, Tokyo, Japan) between January 2024 and January 2025 at Ospedale Papardo, Messina, Italy. We classified lesion complexity a priori using a pragmatic rubric integrating anatomical, surgical, and patient factors. Lesions were categorized as simple or superficial and complex or deep based on the presence of at least two of the following: (1) deep-seated location (e.g., insular, thalamic, or basal ganglia), (2) proximity to or encasement of eloquent cortex or perforating vessels, (3) high vascularity or need for advanced hemostasis, (4) narrow or constrained working corridors requiring extensive brain retraction or trans-sulcal approaches, (5) need for combined skull base or multi-lobar approaches, and (6) prior surgery or radiation. Simple lesions had none of these criteria and complex had two or more. Two attending neurosurgeons independently assigned complexity; disagreements were resolved by consensus. We report raw counts per category and conducted sensitivity analyses pooling moderate and complex lesions due to small sample size (sensitivity analyses pooling moderate and complex lesions were conducted to address sparse cells; effect estimates were compared with the primary three-category analyses to assess robustness). Inclusion criteria were as folllows: patients aged ≥18 years undergoing cranial surgery with exoscope visualization for intra-axial or extra-axial lesions in the supratentorial or infratentorial compartments during the study period. Exclusion criteria were stated as follows: procedures without exoscope use for the intracranial phase, endonasal/transsphenoidal cases, biopsy-only procedures, re-operations within 30 days of an index case, missing key outcome data, and purely vascular or functional procedures not aimed at lesion resection (Table 1).

Patients were categorized into two groups based on surgical complexity: superficial lesions (group 1: n = 13, mean age 53.8 ± 13.3 years) accessible through standard craniotomies (pterional, parietal, frontal, temporo-parietal, frontoparietal, frontotemporal approach) and deep lesions (group 2: n = 13, mean age 59.6 ± 14.3 years) requiring violation of eloquent brain tissue and or/ deep anatomical structures (precuneal interemipheric, suboccipital median, retrosigmoid, parietal, frontal interemispheric transcallosal, temporal, pterional, bicoronal, and fronto-temporo-orbito-zigomatic (Table 2 and Table 3)). The extent of resection was assessed on early postoperative MRI (within 48–72 h) and classified as gross total resection (GTR) no residual enhancement, subtotal resection (STR) residual enhancement, or partial resection, based on neuroradiology reports corroborated by surgeon notes. Complications were recorded through postoperative day 30 using clinical documentation and readmission records. We categorized complications as intraoperative and postoperative [neurological (new focal deficit, seizure, and decline in GCS), medical (infection, thromboembolism, and cardiopulmonary), and surgical (CSF leak, wound dehiscence, and hematoma requiring intervention)]. Complications were also categorized as lesion-related (directly attributable to tumor characteristics and location) or potentially technique-related (possibly influenced by exoscope limitations).

## 3. Results

Gender distribution was equal across groups (7 males, 6–7 females per group). In both groups, all the lesions suspected for glial lesions were administered 5-amino levulinic acid (5-ALA), 6 h before the procedure: superficial lesion cases utilized 5-aminolevulinic acid (5-ALA) fluorescence guidance, while 53.8% of deep lesion cases were administered 5-ALA. The most common pathologies were glioblastoma multiforme (GBM) in both groups, with additional cases including meningiomas, cavernous angiomas, and metastases. Procedural characteristics varied by lesion location. Consistent with prior literature, cases involving deep targets tended to have lower rates of apparent GTR than superficial cases; however, formal statistical comparisons were not performed due to sample size and study design.

Surgical outcomes were assessed by extent of resection (gross total resection (GTR)) vs. subtotal resection (STR) and perioperative complications. Gross total resection was achieved in 100% of superficial lesions compared to 46.2% of deep lesions (*p* < 0.001). Overall complication rates were 14.3% (2/14) for superficial lesions and 23.1% (3/13) for deep lesions. In the superficial group, both complications were intraoperative seizures occurring in patients with frontal lesions (patients 5 and 10), which were classified as lesion-related due to the inherent epileptogenic nature of frontal cortex manipulation. These seizures were successfully managed intraoperatively without permanent sequelae. In the deep lesion group, complications included postoperative hemorrhage in two patients (thalamic cavernous angioma and paratrigonal metastasis) and cranial nerve palsy in one patient with a ponto-mesencephalic angioma. Deep lesion complications were categorized as lesion-related due to complex vascular anatomy and critical location. All the lesion complications appeared to be entirely lesion-related, with the exoscope potentially offering advantages through enhanced visualization for cortical mapping and deep structures visualization. Statistical analysis of 26 neurosurgical cases using the exoscope revealed significant differences between superficial and deep brain tumor resections across multiple clinical parameters. Patient positioning showed a highly significant association with lesion depth (χ^2^ = 26.0, *p* < 0.001), with all superficial lesions (100%) utilizing supine positioning compared to none of the deep lesions (0%), which required non-supine approaches. Gross total resection (GTR) rates demonstrated a statistically significant difference between groups (Fisher’s exact test, *p* = 0.005), with superficial lesions achieving universal GTR success (13/13, 100%) versus deep lesions achieving GTR in only 46.2% of cases (6/13). Complication rates, while numerically higher in the deep lesion group (23.1% vs. 14.3%), did not reach statistical significance (*p* = 0.648) and were all categorized as “postoperative”, suggesting that despite the increased technical complexity, the exoscope did not negatively influence the surgical technique in complex cases. Craniotomy complexity analysis revealed a trend toward more complex surgical approaches in deep lesions (23.1% vs. 0%, *p* = 0.098), though this did not reach statistical significance. These findings provide robust statistical evidence supporting the clinical observation that while superficial lesions benefit from the exoscope’s enhanced visualization in predictable surgical scenarios, deep lesions derive particular value from the system’s 360° rotational capability and flexible positioning, which becomes essential when traditional microscope ergonomics are inadequate for complex anatomical approaches.

## 4. Discussion

This study represents one of the first systematic evaluations of the relationship between craniotomy complexity and surgical outcomes in patients undergoing brain tumor resection with exoscope technology. In this study cohort, the exoscope was effective and safe for resecting both superficial and deep brain lesions, achieving gross total resection rates that were higher in superficial lesions. Despite these differences in resection rates, complication rates were similar between the superficial and deep lesion groups. These results suggest the exoscope performs comparably to traditional operating microscopes, but further prospective studies with long-term follow-up are necessary to evaluate its full oncological impact and establish its best use in neurosurgical oncology. Our analysis of 26 consecutive cases revealed significant differences in surgical complexity between superficial and deep lesions, with deep lesions requiring more complex craniotomy approaches (*p* = 0.022). This finding aligns with established neurosurgical principles, as deep-seated tumors typically necessitate more extensive bone removal, complex surgical corridors, and multiple approach angles to achieve adequate visualization and safe resection [12,13]. The systematic complexity scoring system we developed effectively captured these differences, with deep lesions showing a higher median complexity score compared to superficial lesions. Despite the increased surgical complexity associated with deep lesions, we observed a paradoxical finding regarding gross total resection (GTR) rates. Superficial lesions demonstrated significantly higher GTR rates compared to deep lesions (*p* = 0.001), suggesting that lesion depth and the anatomical site remain critical factors in determining resection extent even with advanced visualization technology. This finding is consistent with previous literature indicating that tumor location and accessibility are primary determinants of the extent of resection [14,15,16]. Importantly, our analysis revealed no significant correlation between craniotomy complexity and complication rates (r = 0.154, *p* = 0.453), nor did we observe significant differences in complication rates between superficial and deep lesion groups (*p* = 0.157). This finding suggests that exoscope technology may provide sufficient visualization quality to maintain safety across different levels of surgical complexity, potentially mitigating some of the traditional risks associated with more complex approaches. The lack of correlation between surgical complexity and complications has important clinical implications for surgical planning and patient counseling. Traditionally, complex approaches for deep seated lesions have been associated with increased morbidity risk [4,17]. Our findings suggest that with appropriate visualization technology, the complexity of the surgical approach may be less predictive of complications than previously assumed. This could influence surgical decision-making, potentially encouraging surgeons to utilize more complex approaches when necessary to optimize tumor exposure and resection.

The superior GTR rates observed in superficial lesions (despite similar complication rates) highlight the continued importance of tumor location in surgical planning. The consistent safety profile observed across different complexity levels may reflect several advantages of exoscope technology. Our results showed that, even in cases of complexity and challenging anatomy, the exoscope manteined same complication rates and an extent of removal in line with the previous literature for the same type of lesions [10,18].

The high-definition 3D visualization provided by modern exoscopes offers superior depth perception compared to traditional 2D monitors, while maintaining the magnification capabilities of operating microscopes [19]. The ergonomic benefits, including natural head positioning and reduced neck strain, may contribute to improved surgical precision during lengthy procedures [20,21]. Additionally, the ability to share the surgical view with the entire operative team through large external monitors may enhance surgical safety through improved communication and collaborative decision-making [2]. This is particularly relevant for complex cases where multiple team members need to understand the surgical anatomy and potential risks.

The consistent safety profile observed across different complexity levels may reflect several advantages of exoscope technology, particularly the revolutionary 360-degree rotational camera capability. The high-definition 3D visualization provided by modern exoscopes offers superior depth perception compared to traditional 2D monitors, while maintaining the magnification capabilities of operating microscopes [7]. The ergonomic benefits, including natural head positioning and reduced neck strain, may contribute to improved surgical precision during lengthy procedures [21].

**Figure 1:**
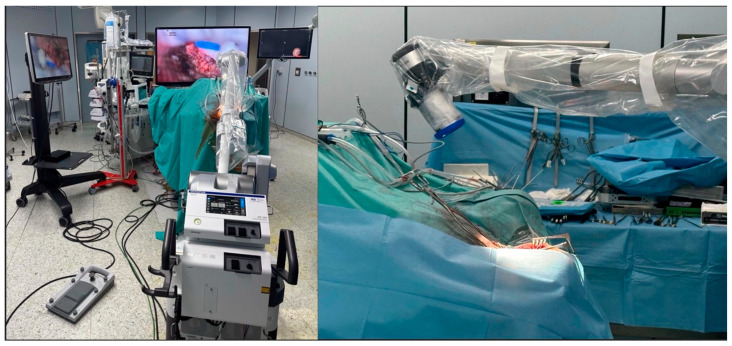
Intraoperative setting. The exoscope was positioned behind the field with the scope 15–20 cm from the site and the monitor facing the surgeon. A frontal 3D 4K display enabled team viewing. This setup ensured surgeon ergonomics, 360° visualization via the telescopic camera, and clear supratentorial views with 30–45° angulation.

The 360-degree rotational functionality of the exoscope camera represents a paradigm shift in neurosurgical visualization, offering unprecedented flexibility in viewing angles without requiring repositioning of the entire optical system [17,19,20,21]. This capability is particularly advantageous in complex craniotomy cases where multiple surgical corridors and approach angles are necessary. Traditional operating microscopes require physical repositioning or tilting of the entire optical assembly to achieve different viewing angles, which can be time-consuming, disruptive to surgical flow, and may compromise sterility. In contrast, the 360-degree rotational camera allows surgeons to seamlessly transition between different viewing perspectives with simple digital controls, maintaining continuous visualization while exploring various aspects of the surgical field. This is especially valuable when working around critical neurovascular structures, where the ability to quickly assess anatomy from multiple angles can enhance safety and surgical decision-making (Figure 1).

Furthermore, the 360-degree rotation eliminates the need for frequent microscope repositioning, which traditionally required interruption of the surgical procedure and potential loss of anatomical orientation. This continuous visualization capability may contribute to reduced operative times and improved surgical efficiency, particularly in complex cases requiring extensive tumor dissection from multiple angles. The rotational camera technology also enhances surgical education and team collaboration by allowing optimal viewing angles to be shared simultaneously with all team members on external monitors [10,20]. This shared perspective can improve communication during critical surgical moments and facilitate real-time consultation with colleagues, regardless of their physical position in the operating room.

This retrospective, single-arm case series was designed as a descriptive feasibility report of early clinical experience using exoscope technology for brain tumor resections involving both superficial and deep locations. Our objective was to document procedural characteristics and key descriptive outcomes, including gross total resection (GTR) and perioperative complications, rather than to test hypotheses or demonstrate superiority over alternative technologies.

Several reports from the literature have stated that GTR rates for superficial cortical tumors commonly range from approximately 70–90% in mixed pathology series, whereas deep-seated lesions often achieve lower GTR rates due to proximity to eloquent structures and vascular constraints [22,23]. As regarding brain tumors amenable to GTR, the gross total resection rate can be as high as around 80–95%. For example, one study reported GTR rates of 80% overall, with some studies showing rates up to 93% or even 100% in selected cases. GTR rates differ by tumor location and surgical techniques. Superficial tumors tend to have higher GTR rates than deep tumors due to accessibility. In glioblastoma and high-grade glioma surgeries, GTR is reported to be achieved in roughly 73–95% of cases depending on localization and intraoperative techniques [24]. Another paper reported GTR achievement of about 55% in a mixed group of brain tumors [25]. Overall complication rates after brain tumor surgery vary widely, commonly reported between 10% and 40% [26]. Neurological deterioration occurs in roughly 10% or more of cases. Local complications (such as wound or local site problems) occur in about 20% or more in some studies. Infratentorial (deep) brain tumors tend to have higher complication rates (~44%) versus supratentorial (more superficial) tumors (~25%) [27].

Against these informal benchmarks, our descriptive outcomes appear directionally consistent for both superficial and deep cases. We emphasize that these comparisons are provided solely to aid interpretability and are not intended as inferential evidence of superiority or noninferiority.

In summary, early experience suggests that exoscope-assisted brain tumor surgery is feasible across a range of case complexities, with descriptive outcomes that align with published ranges for comparable cases. Prospective, adequately powered comparative studies—ideally multicenter and employing standardized outcome definitions—are needed to determine whether exoscope technology confers advantages over traditional microscopy or achieves noninferiority on key clinical endpoints.

Several limitations of our study warrant consideration. It was conducted at a single center with a retrospective design, a small sample size, and limited follow-up, which may constrain generalizability, reduce precision, and preclude definitive conclusions about long-term outcomes and complications. Selection and information biases are possible, and unmeasured confounders may remain despite consistent operative workflows. With these caveats, our findings suggest that exoscopy may be particularly advantageous in cases where surgeon ergonomics and shared visualization can influence performance—such as deep-seated or awkwardly angled surgical approaches, lengthy resections, and teaching settings requiring real-time team situational awareness. The enhanced 3D 4K visualization and flexible angulation also appear useful when wide-angle inspection and frequent line-of-sight adjustments are needed. These clinical implications should be considered hypothesis-generating; validation in larger, multicenter cohorts with longer follow-up is warranted.

The relatively small sample size (n = 26) limits the statistical power to detect smaller effect sizes and may not capture rare complications. The single-center design and specific exoscope system used may limit generalizability to other institutions and technologies. The complexity scoring system, while systematic, remains somewhat subjective and would benefit from validation in larger cohorts. The retrospective nature of our analysis prevents definitive causal inferences about the relationship between exoscope use and outcomes. Limitations are substantial and inform a cautious interpretation. First, this is a single-center, retrospective series with a small sample size, limiting precision and raising risk of type II error. Second, the absence of a concurrent control group using an alternative visualization modality precludes comparative effectiveness inferences. Third, case selection, heterogeneity in pathology and adjunct use (e.g., 5-ALA), and lesion depth inherently confound outcomes and may not reflect broader practice. Fourth, outcomes focused on immediate perioperative events without standardized long-term neurological or oncologic follow-up, and definitions (e.g., the timing and modality of postoperative imaging) may differ from other reports.

Future prospective studies comparing exoscope technology to traditional microscopy across matched complexity levels would provide stronger evidence for the technology’s clinical benefits. Our analysis focused on immediate surgical outcomes and did not evaluate long-term oncological outcomes, quality of life measures, or cost-effectiveness considerations. These factors are crucial for comprehensive technology assessment and should be addressed in future investigations.

## 5. Conclusions

In conclusion, our study demonstrates that exoscope-guided surgery achieved outcomes comparable to those expected with operating microscopes, with high GTR rates in superficial lesions and acceptable results in deep lesions despite greater anatomical complexity, without a significant increase in complications. These findings support the exoscope as a reliable primary visualization platform for both straightforward and complex cranial approaches, with a particular benefit for deep targets where its 360° rotational capability and ergonomics can be advantageous. Larger prospective studies are warranted to confirm these results and refine patient selection.

## Figures and Tables

**Table 1:** Inclusion and exclusion criteria.

Inclusion Criteria	Exclusion Criteria
Adults undergoing brain tumor resection at Ospedale Papardo between January 2024 and January 2025	Non-tumor cranial procedures or biopsy-only cases
Use of exoscope technology (Orbeye Surgical Visualization System)	Repeat operations for the same lesion during the study window
Availability of preoperative imaging and operative records sufficient to classify lesion complexity a priori	Missing key variables precluding complexity classification or outcome assessment
Complete follow-up for primary outcomes	Patients who declined data use or lacked consent
Informed consent obtained for procedure and data use	

**Table 2:** Distribution of patients by lesion site, pathology, and surgical approach (superficial vs. deep): group of patients (n = 26) categorized for superficial (group 1 = 13) and deep (group 2 = 13) lesions; pathology; site of the lesion; and surgical approach.

	Pathology	Site	Craniotomy
Group 1			
**1**	GBM	Frontotemporal left	Pterional
**2**	GBM recurrence	Parietal left	Patietal
**3**	GBM	Parietal right	Patietal
**4**	DNET	Frontal left	Frontal
**5**	Astrocitoma, grade 3	Frontal right	Frontal
**6**	GBM recurrence	Temporoparietal right	Temporo-parietal
**7**	GBM	Frontal right	Frontal
**8**	Glioma IV grado	Frontoparietal right	Frontoparietal
**9**	GBM	Frontoparietal right	Frontoparietal
**10**	Radionecrosis	Frontal right	Frontal
**11**	Metastasis	Frontotemporal right	Frontotemporal
**12**	Astrocitoma grade 2	Frontotemporal right	Frontotemporal
**13**	GBM	Frontal right	Frontal
Group 2			
**1**	Cerebral toxoplasmosis	Paratrigonal right	Precuneal interemipheric
**2**	Meningioma	Tentorium	Suboccipital median
**3**	Cavernous angioma	Thalamo-mesecephalic	Retrosigmoid
**4**	GBM	Parietoccipital left	Parietal
**5**	GBM	Hypothalamus	Frontal interemispheric transcallosal
**6**	GBM	Thalamus	Frontal interemispheric transcallosal
**7**	GBM	Tempo-insular left	Temporal
**8**	GBM	Temporoparieto insular right	Pterional
**9**	GBM	Frontal bilateral with infiltration of Corpus callosum	Bicoronal
**10**	Atypical meningioma	Frontal and intraorbital extraxial Lesion	FTOZ
**11**	Petroclival meningioma	Ceebello pontine angle	Retrosigmoid
**12**	Ponto-mesencefalic angioma	Ponto-mesencefalic region right	Retrosigmoid
**13**	Lung metastasis	Paratrigonal right	Precuneal interemipheric

**Table 3:** Demographic and clinical characteristics: superficial vs. deep.

Characteristic	Superficial Group	Deep Group	Statistical Test	*p*-Value	Significance
Sample Size	13	13	-	nan	-
Age (Years)—Mean ± Sd	49.5 ± 13.5	59.9 ± 13.1	Independent t-test	0.77	Not significant
Age (Years)—Median (Range)	51 (25–67)	61 (36–81)	-	nan	-
Gender—Female N (%)	5 (62.5%)	9 (50.0%)	Fisher’s exact test	0.683	Not significant
Gender—Male N (%)	3 (37.5%)	9 (50.0%)	-	nan	-
Pathology—Gbm/Grade Iv N (%)	5 (62.5%)	9 (50.0%)	Fisher’s exact test	0.683	Not significant
Pathology—Other N (%)	3 (37.5%)	9 (50.0%)	-	nan	-
5-Ala Usage—Yes N (%)	8 (100.0%)	12 (66.7%)	Fisher’s exact test	0.132	Not significant
5-Ala Usage—No N (%)	0 (0.0%)	6 (33.3%)	-	nan	-

## Data Availability

The original contributions presented in this study are included in the article. Further inquiries can be directed to the corresponding author.

## Abbreviations

GTR	gross total resection
STR	subtotal resection
5-ALA	5-aminolevulinic acid
GBM	glioblastoma multiforme
FTOZ	fronto-temporo-orbito-zygomatic
CC	corpus callosum
